# Customized three-dimensional printed ceramic bone grafts for osseous defects: a prospective randomized study

**DOI:** 10.1038/s41598-024-53686-w

**Published:** 2024-02-10

**Authors:** Na-hyun Kim, Byoung-Eun Yang, Sung-Woon On, Ik-Jae Kwon, Kang-Min Ahn, Jong-Ho Lee, Soo-Hwan Byun

**Affiliations:** 1 Department of Conservative Dentistry, Hallym University Sacred Heart Hospital, Anyang, 14066 Republic of Korea; 2 Department of Oral and Maxillofacial Surgery, Hallym University Sacred Heart Hospital, Gwanpyung-ro 170, Anyang, 14066 Republic of Korea; 3 Dental AI-Robotics Center, Hallym University Sacred Heart Hospital, Anyang, 14066 Republic of Korea; 4 Graduate School of Clinical Dentistry, Hallym University, Chuncheon, 24252 Republic of Korea; 5 Institute of Clinical Dentistry, Hallym University, Chuncheon, 24252 Republic of Korea; 6 Department of Oral and Maxillofacial Surgery, Department of Dentistry, Hallym University Dongtan Sacred Heart Hospital, Hwaseong, 18450 Republic of Korea; 7 Department of Oral and Maxillofacial Surgery, School of Dentistry, Seoul National University, Seoul, 03080 Republic of Korea; 8 Department of Oral and Maxillofacial Surgery, Seoul Asan Medical Center, Seoul, 05505 Republic of Korea; 9 Department of Oral and Maxillofacial Surgery, National Cancer Center, Goyang, 10408 Republic of Korea

**Keywords:** Diseases, Medical research

## Abstract

Ridge resorption can result in insufficient bone volume for implant surgery, necessitating bone substitutes to restore the resorption area. Recent advances in computer-aided design and manufacturing enable the use of alloplastic bone graft materials with customizable compositions or shapes. This randomized study evaluated the clinical effectiveness of a customized three-dimensional (3D) printed alloplastic bone material. Sixty patients requiring guided bone regeneration for implant installation following tooth extraction due to alveolar bone resorption were recruited at two institutions. The participants were randomly allocated to either a group that received 3D-printed patient-customized bone graft material or a group that received conventional block bone graft material. Implant installation with bone harvesting was performed approximately 5 months after bone grafting. Histological and radiological assessments of the harvested bone area were performed. The experimental group had a significantly higher percent bone volume and a smaller tissue surface than the control group. Bone volume, bone surface, bone surface/volume ratio, bone surface density (bone surface/total volume), and bone mineral density did not differ significantly between groups. Patient-customized bone graft materials offer convenience and reduce patient discomfort. The findings suggest 3D-printed patient-customized bone graft materials could be used as an alternative for simpler bone grafting procedures.

## Introduction

Treatment of partial and total edentulism using dental implants has become a common method in modern dentistry. A sufficient amount of alveolar bone is essential to successfully install a dental implant^1^. However, alveolar bone atrophy occurs because of periodontal disease, tooth loss, trauma, or tumors, which often results in insufficient bone dimensions for implant installation in a prosthetically ideal position. Therefore, bone augmentation is frequently required to guarantee adequate bone volume^2^. Furthermore, in cases of severe vertical alveolar bone atrophy, implant installation is limited by the surrounding anatomical structures, such as the inferior alveolar nerve in the mandible and the nasal cavity and maxillary sinus in the maxilla^3–5^. Various bone regeneration procedures have been suggested to augment alveolar bone dimensions to obtain a sufficient ridge volume for dental implants^6^. Guided bone regeneration (GBR) was introduced as a treatment method attempting to accomplish bone regeneration, with the application of barrier membranes that mechanically exclude non-osteogenic cell populations from the surrounding soft tissues, allowing osteogenic cell populations derived from the parent bone to occupy the osseous defect^7,8^.

Based on the source material, there are four types of bone graft materials used in dentistry (autogenous, allogenic, xenogenic, and alloplastic). Autogenous bone is considered the gold standard because of its considerable bone-inducing potential and minimal infection risk^9^. Autogenous bone is harvested from a nearby or distant donor site of the same patient. However, it has major disadvantages such as postoperative pain, difficulty in precise application to the defect site, and secondary osseous defects in the donor site^10^. Allografts are derived from a cadaver of the same species and treated to avoid the risk of infection and antigen–antibody reactions. Xenografts originate from non-human sources^11^. To increase biocompatibility, xenografts are manufactured from pure calcium ceramic, from which all organic components have been eliminated. Graft materials derived from living organisms may carry the risk of illness transmission and immune reactions. Accordingly, these materials are treated with X-rays, freezing, and chemicals to prohibit this, thereby lowering the potential for osteogenesis. However, despite these treatments, there remains a risk of prion contamination in allografts and xenografts. The most widely used xenograft material is deproteinized bovine bone minerals, Bio-Oss (Geistlich Pharma AG, Wolhusen, Switzerland). Alloplasts use completely synthetic bone graft material not obtained from living organisms. Calcium and other elements are mixed in various materials, such as hydroxyapatite, tricalcium phosphate, bioactive glass, and calcium sulfate^12^. These four types of graft materials can differ in porosity (dense, macroporous, microporous) and shape (crystalline, amorphous)^13^. The combined use of bone graft materials depends on the size and topography of the bony defect. Particle bone products are easy to apply to the defect site, but fixation and stability often pose challenges. In contrast to particulate materials, block grafts have the advantage of easy and stable fixation using osteosynthesis screws^14^. There is an increasing demand for the development of customized block bone graft materials that can be easily used and precisely applied regardless of the defect size^15,16^.

Computer-aided design/computer-aided manufacturing (CAD/CAM) technology allows customized fabrication of alloplastic bone materials for sophisticated alveolar ridge augmentation procedures^17–20^. Numerous case studies have proved the high-precision fit and successful application of customized allograft bone graft materials^21^. The advantage of using such graft materials is the ability to customize bone graft materials using CAD/CAM technology for making alloplastic bone blocks that ideally match the geometry of the defect^22,23^. By using this technique, autologous bone harvesting and manual modification of the graft can be avoided, saving time and minimizing the risk of contamination and complications. Additionally, the contact area between the bone block and the bone defect is maximized due to the excellent fit of the graft materials, resulting in optimal graft integration^24,25^.

This study aimed to evaluate the clinical effectiveness of patient-customized digital bone grafting materials using 3D printing, compared with the conventional block bone graft material, in patients with severe alveolar ridge atrophy. This randomized controlled trial was blinded, involving experimental and control groups at two different institutions.

## Results

**Figure 1:**
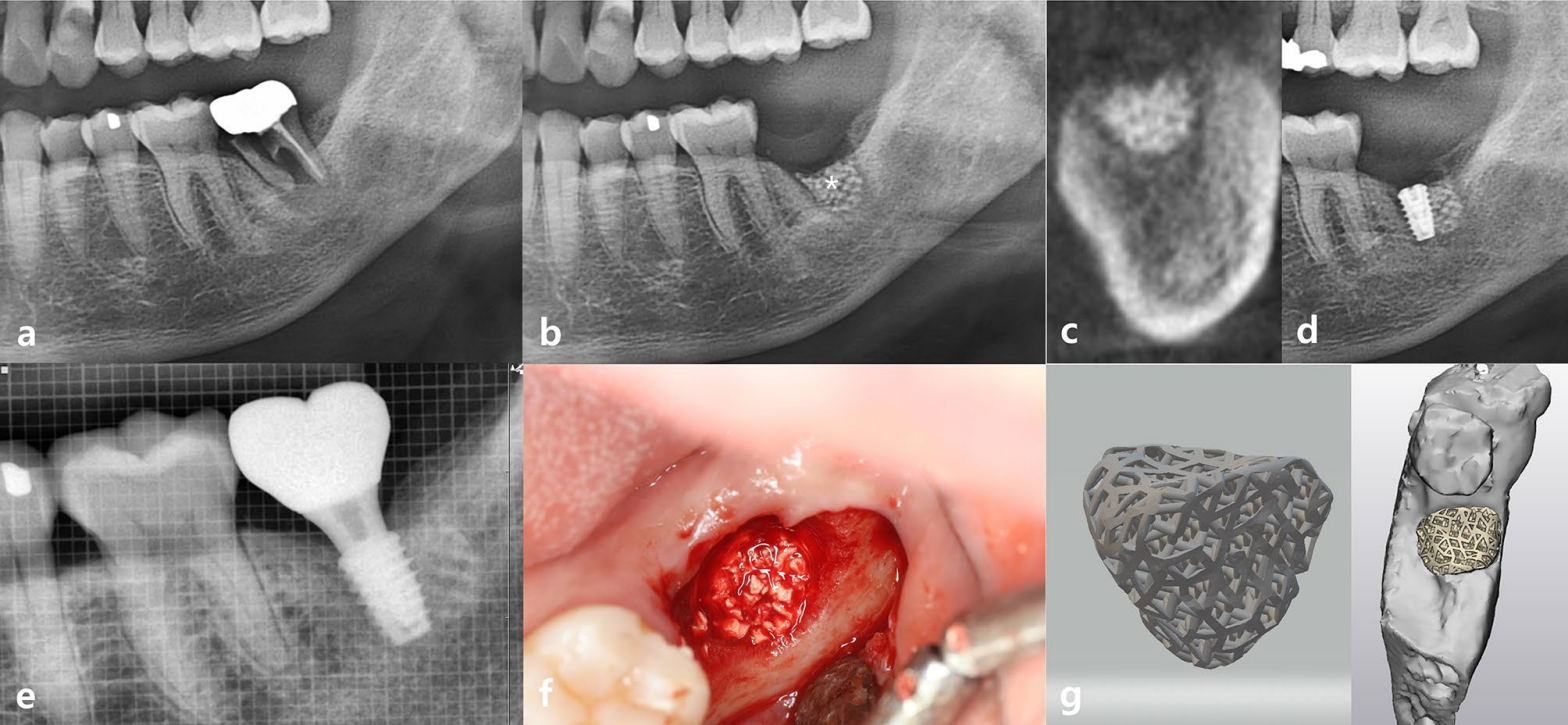
Serial clinical and radiographic images of the experimental group receiving customized 3D-printed ceramic bone grafts. (**a**) preoperative panoramic radiograph of tooth #37; (**b**) postoperative panoramic radiograph after surgical extraction of tooth #37 with GBR using OSTEON 3D and Collagen Membrane; white asterisk indicates grafted bone material; (**c**) CBCT image before the implant placement at visit 5; (**d**) panoramic radiograph after implant fixture installation; (**e**) panoramic radiograph after final implant final prosthesis; (**f**) clinical photo of bone grafting; (**g**) 3D digital image of design for fabrication of customized 3D printed ceramic bone grafts (OSTEON 3D).

**Figure 2:**
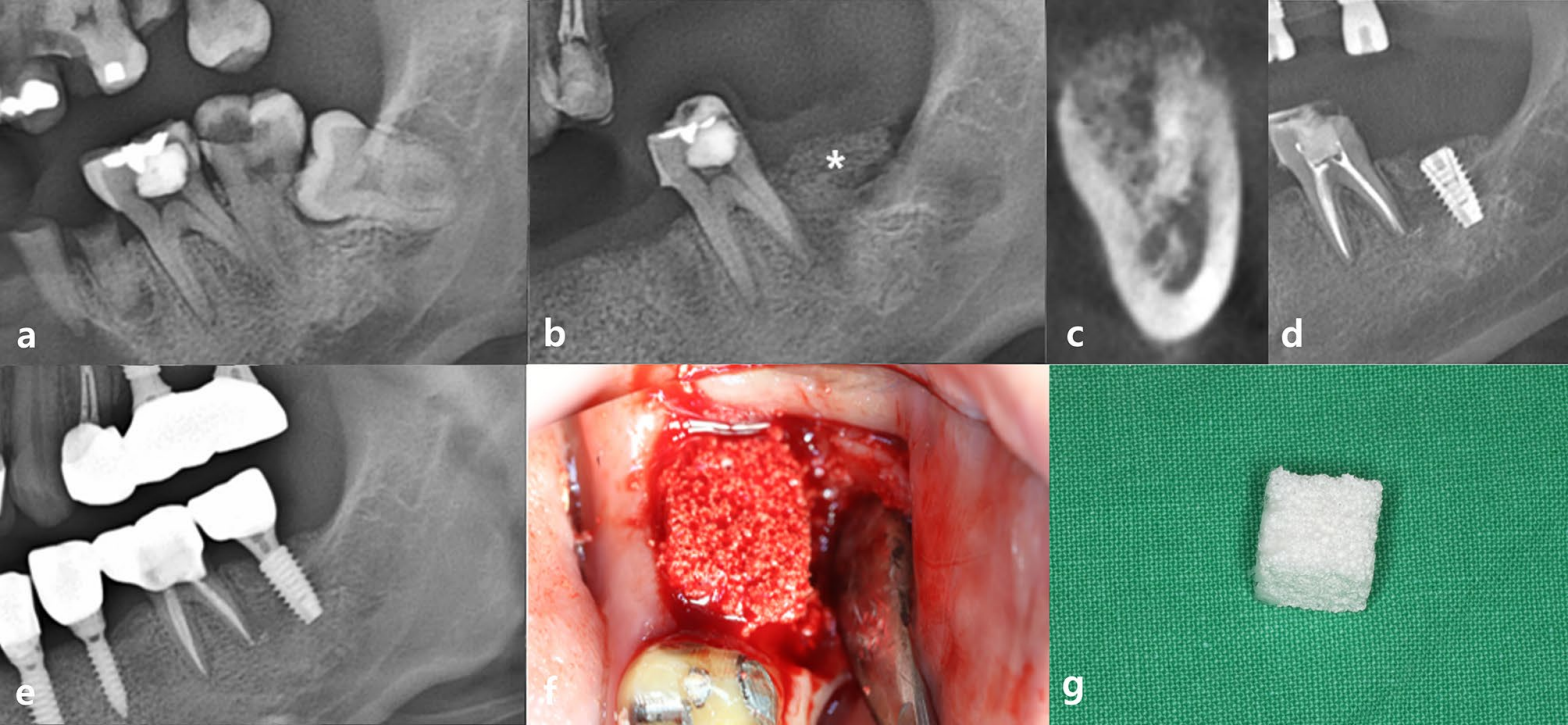
Serial clinical and radiographic images of the control group receiving conventional block type bone grafts. (**a**) preoperative panoramic radiograph of tooth #37; (**b**) postoperative panoramic radiograph after surgical extraction of tooth #37 with GBR using OSTEON 3 block and Collagen Membrane; white asterisk indicates grafted bone material; (**c**) CBCT image before the implant placement at visit 5; (**d**) panoramic radiograph after implant fixture installation; (**e**) panoramic radiograph after final implant final prosthesis; (**f**) clinical photo of bone grafting; (**g**) conventional block-type bone graft material (OSTEON 3 block).

Representative clinical and radiographic images of the experimental and control groups are shown in Figs. 1 and 2, respectively. Figure 1 shows a representative case of the experimental group. The patient had a fractured left lower second molar (Fig. 1a). The tooth was extracted, and the defect was filled with a customized 3D-printed bone graft simultaneously (Fig. 1b). An implant fixture was placed at the site after five months (Fig. 1d). The implant prosthesis was connected three months after fixture placement, and the implant was well maintained in the area (Fig. 1e). Figure 1f shows the clinical photograph of bone grafting, and Fig. 1g shows the 3D design of the defect customized bone grafts. Figure 2 shows a representative case of the control group. The left lower second molar had severe dental caries (Fig. 2a). The left lower second molar and the third molar were removed, and the conventional block bone grafting was performed at the second molar area simultaneously (Fig. 2b). A fixture was placed at the second molar area after five months (Fig. 2d). The final prosthesis was delivered three months after the fixture placement (Fig. 2e). Figure 2f shows the clinical photograph of bone grafting, and Fig. 2g shows the conventional block bone grafts. Both the experimental and control groups underwent successful dental rehabilitation through bone grafting and implant installation without any issues. In both groups, implant fixture placement was performed approximately 5 months after extraction and GBR. No participants reported any specific discomfort after the surgery.

**Figure 3:**
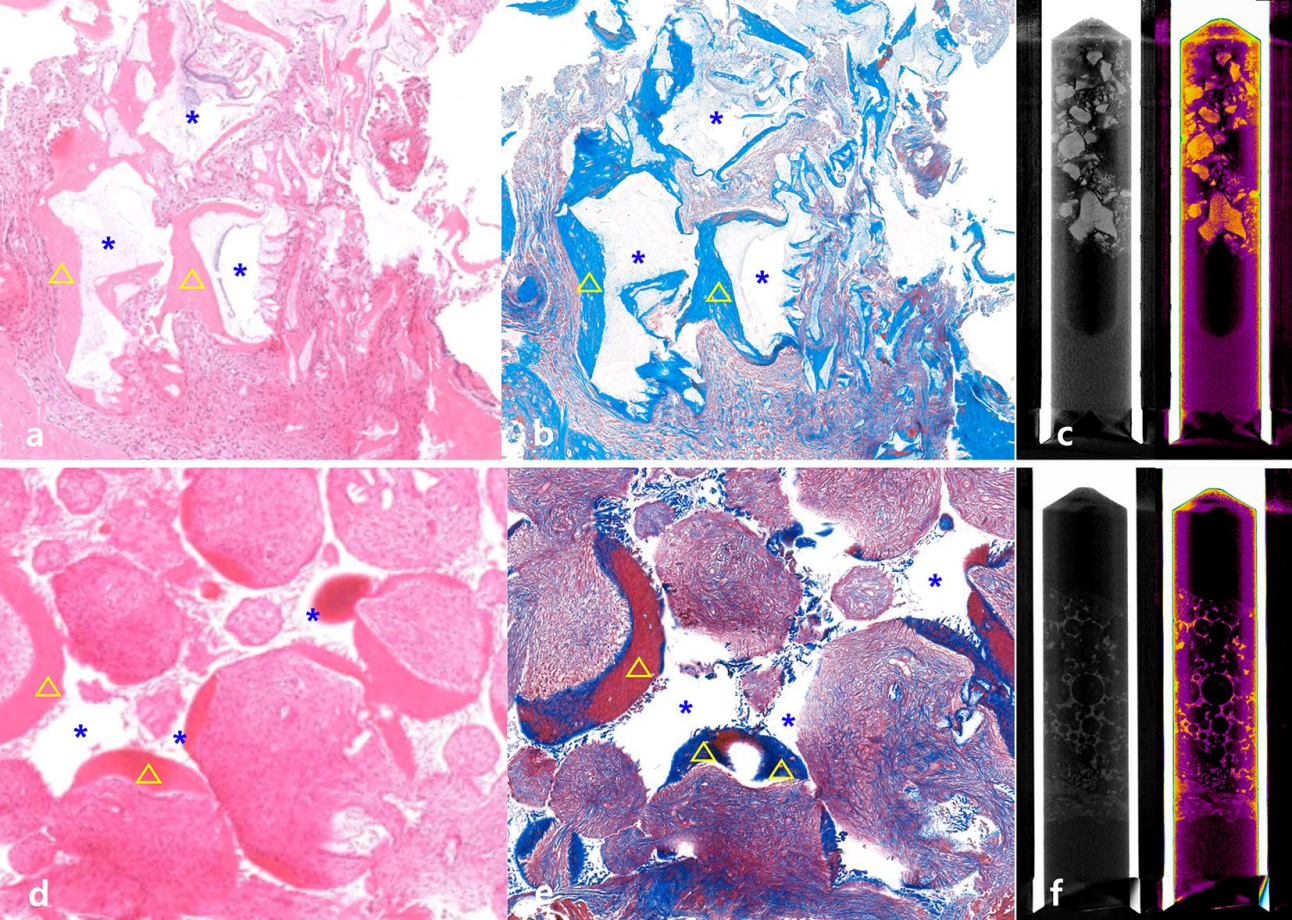
Histological and radiological examination. (**a**) Hematoxylin and eosin (H&E) staining of harvested bone from the experimental group; (**b**) Masson’s trichrome staining of harvested bone from the experimental group; (**c**) Micro-CT examination of harvested bone specimen in the experimental group; (**d**) H&E staining of harvested bone from the control group; (**e**) Masson’s trichrome staining of harvested bone from the control group; blue asterisk indicates grafted bone material; yellow triangle indicates newly formed bone; (**f**) Micro-CT examination of harvested bone specimen in the control group.

Histological and radiological analyses revealed newly formed bone and residual bone graft material in both groups. Histological and radiological images of the experimental and control groups are shown in Fig. 3. Figure 3 shows the proper new bone formation in both groups. Both groups had new bone formation (triangle) around the grafted bone materials (asterisk). There was no specific inflammatory response around the graft materials in both groups.

Table 1 presents the comparison results of various parameters related to tissue and bone characteristics between the control group and the experimental (OSTEON 3D) group. The parameters measured include tissue volume (TV), bone volume (BV), percent bone volume (BV/TV), tissue surface (TS), bone surface (BS), bone surface/volume ratio (BS/BV), bone surface density (BS/TV), and bone mineral density.

**Table 1:** Comparison of parameters related to tissue and bone characteristics between the control and experimental groups.

	Group	Mean	SD	*P* value
Tissue volume (TV) (mm^3^)	Control	50.227	11.507	0.052
	Exp	44.869	5.593	
Bone volume (BV) (mm^3^)	Control	8.116	1.608	0.173
	Exp	8.938	2.317	
Percent bone volume (BV/TV) (%)	Control	16.350	3.062	0.049*
	Exp	20.012	7.831	
Tissue surface (TS) (mm^2^)	Control	96.451	12.661	0.005*
	Exp	84.897	15.041	
Bone surface (BS) (mm^2^)	Control	761.254	186.069	0.228
	Exp	682.471	223.076	
Bone surface/volume ratio (BS/BV) (1/mm)	Control	104.523	23.482	0.122
	Exp	117.115	32.715	
Bone surface density (BS/TV) (1/mm)	Control	15.869	4.523	0.559
	Exp	14.947	6.390	
Bone mineral density (g/cm^3^)	Control	0.957	0.416	0.131
	Exp	0.766	0.459	

The mean BV/TV was higher in the Osteon 3D group than in the control group, whereas the mean TS was lower in the Osteon 3D group than in the control group.

## Discussion

Clinicians encounter a relatively large number of patients who require implant placement along with bone grafting. The loss of a single tooth or multiple teeth may result in a substantial deficit of the residual alveolar ridge. Such situations require augmentation of lost bony structures to provide optimal conditions for dental implant placement and subsequent prosthetic rehabilitation. Currently, digital technology allows dentists to design and fabricate custom-made synthetic bone graft materials digitally for use in bone reconstructive procedures. Several studies in various dental fields have shown that combining modern image acquisition techniques with 3D reconstruction software allows clinicians to obtain custom-made bone graft materials. This combination enables dentists to virtually plan the reconstruction of an atrophic area of bone on a computer and fabricate a patient-customized bone graft material by designing its size, thickness, and shape. The patient-specific bone graft material can be fabricated by milling or 3D printing synthetic bone substitutes that mimic the structure of natural bone and, therefore, promote the formation of new bone when implanted^26,27^.

Recent developments in design and fabrication technologies have made it possible to design 3D scaffolds with controlled architecture using computational modeling and simulations. The present controlled, randomized clinical study evaluated 3D patient-customized bone graft material OSTEON 3D in clinical practice. The use of CAD/CAM and 3D printing for digital reconstruction and graft fabrication in alveolar ridge augmentation procedures can provide significant benefits to both patients and clinicians^28^. This clinical trial highlighted both the benefits and challenges associated with 3D-printed bone materials. First, it allowed for detailed preoperative planning and design of the desired final grafting outcome and virtual evaluation of the desired outcome relative to the final prosthetic reconstruction. Second, it had the potential to generate customized grafts with the best adaptation. Third, it allowed for significant intra-operative time reduction; less surgical time typically results in more uneventful healing, less patient discomfort, and an overall better patient experience^29^. However, there were some disadvantages to customized, 3D-printed bone materials. First, the modeling process for 3D printing began with identifying the defect size on cone-beam computed tomography (CBCT) images. However, the accuracy of CBCT imaging was questionable, and assessing the presence or absence of bone in CBCT could be inaccurate. Thus, effective communication between design teams and clinicians was essential, and accurate CT reference value settings were crucial. Second, the real defect size and shape of the host bone might not exactly accommodate the 3D-printed bone graft due to residual granulation tissue and complex anatomical structures. Moreover, the lack of bone formation due to micro-movements caused by microscopic gaps between an inaccurate customized bone graft and the septum area can hinder osteogenesis^30^. Third, a challenge in clinical application arises due to the brittleness of the 3D-printed bone materials employed in this study; unlike the allogenic block bone from other research, which offers greater strength and can be securely anchored with screws, the 3D-printed bone materials present fixation challenges. Given their block-like nature, achieving reliable fixation is crucial for addressing bone defects. This difficulty in securing the 3D-printed bone materials represents a significant clinical limitation, highlighting the imperative to enhance their strength for effective application in defect areas.

The present study conducted a histological analysis of new bone formation and residual grafts using eight parameters. The experimental group had a significantly higher BV/TV and lower TS than the control group. However, no significant differences were noted between the experimental and control groups in BV, BS, the BS/BV ratio, BS density (BS/TV), or bone mineral density. Based on these parameters, it can be concluded that bone formation was better in the experimental group, but the difference was not substantial. This outcome might stem from the fact that similar ingredients were used in both groups, with the primary distinction being the customized 3D-printed material of the experimental group versus the non-customized block grafts of the control group. Particularly, no notable differences were observed in the implant installation results in the bone grafting site from a clinical perspective. Thus, these results could suggest that there were no specific clinical differences in the histological regenerative capacity of new bone formation between the experimental and control groups.

Kijartorn et al.^31^ reported no statistically significant differences in alveolar ridge resorption following alveolar ridge preservation with 3D-printed ceramic bone grafts compared with conventional particle-type bone graft materials. In a related study, Carolina et al. compared 3D-printed ceramic block grafts and autogenous block grafts for atrophic maxilla rehabilitation^32^. Notably, the literature on the distinctions between 3D-printed block-type ceramic grafts and conventional block-type ceramic grafts is limited, with most previous clinical studies focusing on comparisons with particle-type bone grafts or autogenous block bone grafts^31–33^. This study’s clinical significance lies in its exploration of the effectiveness of customized 3D bone grafting techniques, particularly in the context of block-type ceramic grafts. Unlike the majority of prior research, which predominantly contrasted 3D printed ceramic grafts with particle-type bone or autogenous block bone, this study contributes valuable insights into a less-explored aspect of bone grafting. Importantly, existing literature consistently suggests that patient-specific bone grafts have the potential to enhance patient satisfaction, reducing the reliance on autogenous bone grafts and thereby mitigating associated implications such as patient morbidity. The findings from this study thus provide a nuanced perspective on the clinical benefits of customized 3D bone grafting techniques.

If clinicians use patient-customized bone graft materials during bone grafting, there is no need to trim the conventional block bone to fit the defect size. As mentioned above, since there was no clinical difference in bone regeneration capacity, using patient-customized bone graft material offers several advantages for clinicians, such as convenience, reduced patient discomfort, and lower potential for complications. Therefore, patient-customized bone graft materials have more benefits in terms of convenience and efficiency than conventional block-shaped bone graft materials.

The limitation of this study is that the size and morphology of bone defects varied among patients, resulting in different levels of natural bone formation after using patient-customized or conventional bone graft materials. In addition to the variability in the size and morphology of bone defects among patients, several other factors may have influenced the outcomes of the study. Patient-specific factors such as age, overall health, and pre-existing medical conditions could have affected the natural bone formation process. Moreover, the surgical technique used for graft placement, the type of bone defects, and the postoperative care protocols used may have contributed to the observed differences in bone regeneration. Furthermore, long-term follow-up assessments are crucial to evaluate the durability and stability of bone regeneration over time. Factors such as graft integration, implant success rates, and patient-reported outcomes should be assessed to provide a more comprehensive understanding of the overall efficacy and sustainability of the bone grafting procedures under investigation. Future research could consider implementing a more standardized approach to address these potential confounding factors, including a larger sample size with more homogenous patient populations. This would enable a more comprehensive analysis of the effectiveness of patient-customized versus conventional block bone graft materials while minimizing the impact of individual variations.

## Conclusions

The results of this study hint at the potential of 3D-printed patient-customized bone graft materials as an alternative for simplified bone grafting procedures. However, these materials did not demonstrate significant bone regeneration capability compared to conventional graft materials. Therefore, further research is necessary to comprehensively evaluate and enhance the bone regeneration potential within these materials.

## Methods

### Study participants

In this prospective randomized clinical study, we recruited 60 patients needing implant treatment for suspected osseous defects, with 30 recruited from Seoul National University Dental Hospital and 30 from Hallym University Sacred Heart Hospital. The participants comprised 28 men and 32 women (mean age, 57.5 years; range, 23–77 years). This study was conducted in accordance with the guidelines of the Declaration of Helsinki and approved by the Institutional Review Board (IRB) of Hallym University Sacred Heart Hospital (IRB No. 2020-06-020-023; 17/11/2020) and Seoul National University Dental Hospital (IRB No. CDE21001; 11/06/2020). Personal information was not disclosed throughout the entire study and publication process. Informed consent was obtained from all participants and/or their legal guardians. The inclusion and exclusion criteria are presented in Table 2.

**Table 2:** Inclusion and exclusion criteria.

Inclusion criteria
(1) Adult patients aged 20–74 years in whom maxillary and mandibular growth was complete (2) Patients with one or more missing teeth who planned to receive dental implant treatment with bone grafting (3) Patients who did not smoke or smoked < 20 cigarettes per day (4) Patients who agreed to participate in the clinical trial and signed consent forms
Exclusion criteria
(1) Pregnant patients (2) Patients with uncontrolled systemic metabolic diseases (e.g., diabetes, hypertension) (3) Patients who continuously took medicines that may affect bone metabolism for 7 days or longer within the prior 6 months (e.g., bisphosphonates, corticosteroids) (4) Patients with uncontrolled gingivitis, periodontitis, or dental caries (5) Patients who received radiation therapy at the treatment site (6) Patients who required anticoagulants for uncontrolled bleeding disorders (7) Patients who were allergic to bone graft materials and implant materials (8) Patients who smoked more than 20 cigarettes per day (9) Patients for whom researchers deemed that participation in the clinical trial was inappropriate as it may affect other ethical or clinical trial results

A total of 30 patients from each dental hospital were randomly classified into an experimental group of 15 patients and a control group of 15 patients. A statistical program was used to assign patients in this clinical trial to enhance comparability between the experimental and control groups and prevent bias in their allocation. The allocation table was managed by the designated research personnel appointed by the principal investigator, who recorded and maintained the subject identification codes. A customized bone graft material OSTEON 3D (Dentium, Seoul, Korea) was used in the experimental group, and the conventional bone graft material OSTEON3 block (Dentium) was used in the control group for alveolar bone augmentation.

### Materials

The composition of the OSTEON 3D and OSTEON3 block materials was mostly similar, with the difference being that OSTEON 3D was customized to fit the defect site using 3D printing. In OSTEON3 block materials, the pore size was 200–400 µm, and the porosity was approximately 80%. The OSTEON 3D group provides synthetic and alloplastic bone graft materials that can be tailored to specific defect conditions. The pore size and porosity can be adjusted and were measured to be between 0.7 and 1.2 mm and 70–80%, respectively. The OSTEON 3D and OSTEON3 blocks are composed of a mixture of hydroxyapatite and β-tricalcium phosphate in a 60:40 ratio and have particle sizes ranging from 0.2 to 2.0 mm in block form.

### Fabrication of patient-customized bone graft materials

**Figure 4:**
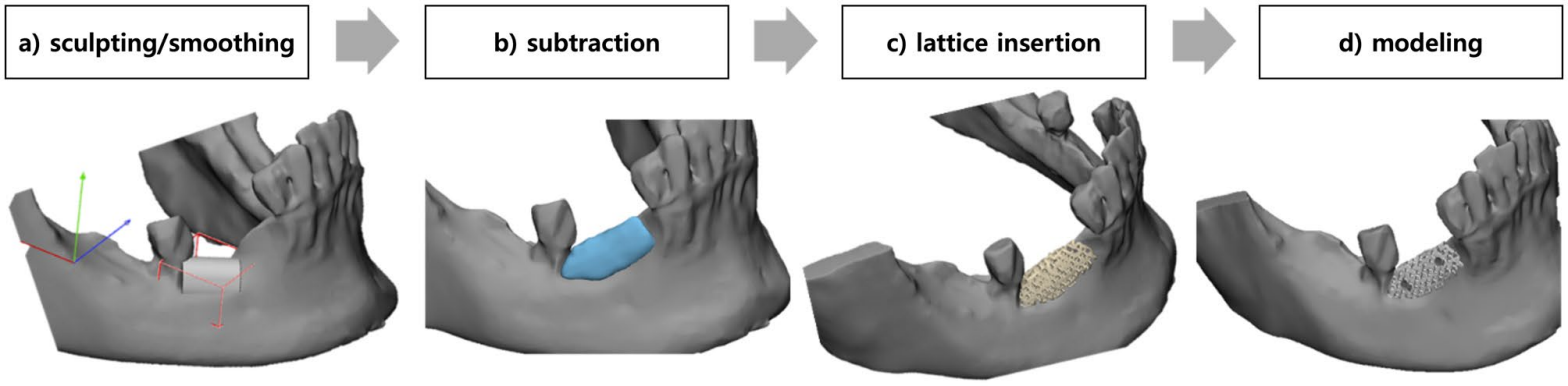
Defect modeling procedure using cone-beam computed tomography data. (**a**) sculpting/smoothing; (**b**) subtraction; (**c**) lattice insertion; (**d**) modeling.

The OSTEON 3D material was produced by calculating the defect size using computer software based on CBCT data obtained at the first visit. Materialise Mimics (Materialise, Leuven, Belgium) and 3-Matic (Materialise) were used for the calculation and design of customized bone grafts, respectively. The OSTEON 3D material was modeled with high precision by predicting the succeeding implant position and the final state of the bone reconstruction. Using CBCT data, defect modeling proceeded in the order of sculpting/smoothing, subtraction, lattice insertion, and modeling (Fig. 4). Next, the OSTEON 3D material was 3D printed using digital light processing, and sintering was performed at 1200 °C. The OSTEON 3D material was modified based on communication between clinicians and technicians during this process. A mixture of hydroxyapatite and β-tricalcium phosphate (60:40 wt%), which are the main components of OSTEON 3D, was produced using 3D printing, and the final product was stored at room temperature, avoiding direct sunlight.

### Schedule of visits

This study was conducted over a total of five visits for each patient. Detailed information for each visit is presented in Table 3.

**Table 3:** Detailed information of patient visits.

Day	Visit 1	Visit 2	Visit 3	Visit 4	Visit 5
	Screening	0	2 weeks (± 1 week)	3 months (± 1 month)	5 months (± 2 months)
Consent to participate in clinical trials	O				
Check demographic information, medical/dental history, inclusion/exclusion criteria	O				
Application of customized 3D-printed patient- bone graft material (OSTEON 3D)		O			
Panoramic radiography	O	O		O	O
Cone-beam computed tomography	O	O			O
Clinical photography	O	O	O	O	O
Adverse reaction evaluation	O	O	O	O	O
Clinical evaluation			O	O	O
Bone harvesting for histological analysis					O

### Surgical procedure using patient-customized bone graft materials

All patients had teeth for which extraction with implant surgery was recommended for rehabilitation. Bone grafting was essential for subsequent implant installation as all patients had severe alveolar bone atrophy. By evaluating the CBCT data, patient-customized OSTEON 3D bone grafts were manufactured via 3D printing. One week after tooth extraction, GBR was performed using OSTEON 3D and Collagen Membrane (Dentium). The procedure was performed under local anesthesia with 2% lidocaine containing 1:100,000 epinephrine. The bone graft material was delicately pushed in to fit the defect as accurately as possible. Collagen Membrane can be used when primary closure is possible, as the product has a thin single layer of porcine collagen to prevent wound dehiscence. Figure-of-eight sutures using Dafilon 4-0 (B. Braun, Melsungen, Germany) were used for wound closure. Implant installation was performed 5 months (± 2 months) after GBR. At the time of bone drilling for fixture placement, grafted bone was collected using a trephine bur with a diameter of 2 mm, and the collected bone was histologically analyzed. Next, impressions for implant prostheses were obtained at the Department of Prosthetics, and the final prosthesis was completed approximately 1.5 months after the second implant surgery. The entire procedure of bone grafting, fixture placement, and bone collecting was performed by one specialist, an oral and maxillofacial surgeon specialist from each hospital.

### Histological analysis

This study conducted histological analysis using hematoxylin and eosin staining and Masson's trichrome staining. The samples were fixed in 10% neutral buffered formalin and embedded in paraffin blocks for hematoxylin and eosin staining. These blocks were then sectioned to a thickness of 4 μm. Following this, sections underwent deparaffinization and hydration using an ethyl alcohol gradient, ranging from 100 to 70%. After washing in water, Mayer’s hematoxylin solution was applied for 10 min. Following another 10 min washing step, 1% alcoholic Eosin Y (Sigma-Aldrich, St. Louis, MO, USA) was applied for 10 min. Subsequently, a series of ethanol washes were performed, including 70% EtOH (1 wash), 95% EtOH (1 wash), and 100% EtOH (2 washes). Finally, slides were mounted with Permount Mounting Medium (Electron Microscopy Sciences, Hatfield, PA, USA) using coverslips. For Masson's trichrome staining, the samples followed a similar procedure. They were initially fixed in 10% neutral buffered formalin and subsequently decalcified using 10% formic acid. After embedding in paraffin blocks, the samples were sectioned at a thickness of 3 μm, followed by deparaffinization and hydration using an ethyl alcohol gradient ranging from 100 to 70%. Following a thorough wash in tap water, Weigert’s iron hematoxylin was applied for 5 min, succeeded by a 10 min washing step, and incubated in Biebrich Scarlet solution for 3 min. Subsequently, a 2 min differentiation using 3% phosphomolybdic–phosphotungstic acid was carried out and Aniline blue was applied for 5 min. After washing in tap water, samples were treated with 1% acetic acid for 1 min, followed by another quick wash. The sections then went through a graded ethanol series: 70% EtOH (1 wash), 95% EtOH (1 wash), and 100% EtOH (2 washes). Finally, the slides were mounted with Permount Mounting Medium using coverslips.

### Radiological analysis

The specimen was measured using Micro-CT (SkyScan1173; Bruker-CT, Kartuizersweg 3B 2550 Kontich, Belgium). SkyScan1173 control software (ver 1.6, Bruker-CT) was used for obtaining the measurements, with a tube voltage of 130 kVp, a tube current of 60 μA, 1 mm aluminum filtration (Filter), an exposure time of 500 ms, (2240 × 2240) pixels, and a pixel size of 7.14 μm. The rotation angle was rotated by 0.3˚ and 180˚ to obtain 800 high-resolution images. For cross-sectional reconstruction, an image of 2240 × 2240 pixels was obtained using Nrecon (ver 1.7.0.4, Bruker-CT), and the cross-sectional image was aligned using Dataviewer (ver 1.5.1.2, Bruker-CT). For data analysis, CTAn (ver 1.17.7.2, Brooker-CT) was used to set the inside of the Trephine drill as an area, and the volumes of the nephrotic area and this parameter were analyzed by setting the threshold to 45–61 for the amount of bone present in the area. Bone mineral density was obtained using Bruker’s standard sample phantom and applied to the specimen to be analyzed. Bone mineral density was analyzed using CTAn (ver 1.17.7.2, Bruker-CT).

### Statistical analyses

SPSS (v.20, IBM Corp., Armonk, NY, USA) was used for statistical analysis. Data are represented as mean ± standard deviation. Independent sample t-tests were used to compare variables between the two groups. *P* values less than 0.05 were considered statistically significant.

## Data Availability

The datasets used and/or analyzed during the current study are available from the corresponding author upon reasonable request.
